# Declines in violence and police arrest among female sex workers in Karnataka state, south India, following a comprehensive HIV prevention programme

**DOI:** 10.7448/IAS.18.1.20079

**Published:** 2015-10-16

**Authors:** Tara S Beattie, Parinita Bhattacharjee, Shajy Isac, HL Mohan, Milena Simic-Lawson, BM Ramesh, James F Blanchard, Stephen Moses, Charlotte H Watts, Lori Heise

**Affiliations:** 1 Department of Global Health and Development, London School of Hygiene & Tropical Medicine, London, UK; 2 Karnataka Health Promotion Trust, Bangalore, India; 3 Department of Community Health Sciences, University of Manitoba, Winnipeg, Canada; 4 Department of Medical Microbiology, University of Manitoba, Winnipeg, Canada

**Keywords:** female sex work, violence, arrests, alcohol, HIV, India, HIV prevention, structural drivers

## Abstract

**Introduction:**

Female sex workers (FSWs) frequently experience violence, harassment and arrest by the police or their clients, but there is little evidence as to the impact that such factors may have on HIV risk or whether community interventions could mitigate this impact.

**Methods:**

As part of the evaluation of the Avahan programme in Karnataka, serial integrated behavioural and biological assessment (IBBA) surveys (four districts) (2005 to 2011) and anonymous polling booth surveys (PBS) (16 districts) (2007 to 2011) were conducted with random samples of FSWs. Logistic regression analysis was used to assess 1) changes in reported violence and arrests over time and 2) associations between violence by non-partners and police arrest and HIV/STI risk and prevalence. Mediation analysis was used to identify mediating factors.

**Results:**

5,792 FSWs participated in the IBBAs and 15,813 participated in the PBS. Over time, there were significant reductions in the percentages of FSWs reporting being raped in the past year (PBS) (30.0% in 2007, 10.0% in 2011, *p<*0.001), being arrested in the past year [adjusted odds ratio (AOR) 0.57 (0.35, 0.93), *p=*0.025] and being beaten in the past six months by a non-partner (clients, police, pimps, strangers, rowdies) [AOR 0.69 (0.49, 0.95), *p=*0.024)] (IBBA). The proportion drinking alcohol (during the past week) also fell significantly (32.5% in 2005, 24.9% in 2008, 16.8% in 2011; *p<*0.001). Violence by non-partners (being raped in the past year and/or beaten in the past six months) and being arrested in the past year were both strongly associated with HIV infection [AOR 1.59 (1.18, 2.15), *p=*0.002; AOR 1.91 (1.17, 3.12), *p=*0.01, respectively]. They were also associated with drinking alcohol (during the past week) [AOR 1.98 (1.54, 2.53), *p<*0.001; AOR 2.79 (1.93, 4.04), *p<*0.001, respectively], reduced condom self-efficacy with clients [AOR 0.36 (0.27, 0.47), *p<*0.001; AOR 0.62 (0.39, 0.98), *p=*0.039, respectively], symptomatic STI (during the past year) [AOR 2.62 (2.07, 3.30), *p<*0.001; AOR 2.17 (1.51, 3.13), *p<*0.001, respectively], gonorrhoea infection [AOR 2.79 (1.51, 5.15), *p=*0.001; AOR 2.69 (0.96, 7.56), *p=*0.060, respectively] and syphilis infection [AOR 1.86 (1.04, 3.31), *p=*0.036; AOR 3.35 (1.78, 6.28), *p<*0.001, respectively], but not with exposure to peer education, community mobilization or HIV testing uptake. Mediation analysis suggests that alcohol use and STIs may partially mediate the association between violence or arrests and HIV prevalence.

**Discussion:**

Violence by non-partners and arrest are both strongly associated with HIV infection among FSWs. Large-scale, comprehensive HIV prevention programming can reduce violence, arrests and HIV/STI infection among FSWs.

## Introduction

Female sex workers (FSWs) are at disproportionally high risk of acquiring HIV infection [1]. Arrests and violence against them by clients, pimps or the police are important daily concerns [2]. As well as being an abuse of their rights, exposures to violence can undermine HIV prevention efforts, either directly through forced sex with an HIV-positive perpetrator or indirectly through decreased ability to negotiate condom use or pursue other protective behaviours [3]. In addition, men who are violent against women are more likely to exhibit a clustering of risk behaviours, such as multiple sexual partners, increased alcohol use, unprotected anal sex and low/no condom use – behaviours that put them, and therefore their sexual partners, at increased risk of HIV/STIs [3]. The clandestine/illegal status of sex work and the stigmatization of women who sell sex may also mean that violence against FSWs goes unreported; violent behaviour, as well as the risk of arrest and incarceration, can thus continue unchecked [4–6]. The fear of violence and/or violence-induced mental health problems (such as depression, anxiety and alcohol use) may also reduce FSWs’ ability to negotiate condom use or to access HIV and STI testing and treatment services [4, 7–11], with likely increases in HIV incidence [12]. Alcohol use is common among sex worker populations and is frequently used as a maladaptive coping mechanism [9]. Violence exposure may lead to increased alcohol consumption as women struggle to cope with their experiences or, conversely, alcohol use may lead to police arrest or violence from clients if intoxicated sex workers become abusive [13, 14]. Alcohol use among FSWs is associated with a host of risk factors for HIV, including sexual violence, unprotected sex, group sex, anal sex, sex while unconscious, sex during menses and unprotected anal sex [15–19]; it is also associated with HIV incidence [20].

The police can act as both protectors from and perpetrators of violence. Police harassment and arrest can increase vulnerability to HIV/STIs through multiple mechanisms [2]. The fear of a police raid or arrest can stop women from carrying condoms and drive sex work underground, forcing women to work in more perilous settings and to agree to unprotected sex [21]. Police extortion, in the form of demands for (unprotected) sex in return for non-arrest, is associated with inconsistent condom use and STI symptoms [22] and can lead women to take on riskier clients and riskier types of sex [4, 23, 24]. Sexual violence during arrest or while being held in detention centres can directly increase HIV/STI risk, with arrest and detention serving as a context for police harassment, mistreatment and physical and sexual violence [25]. Finally, the substantial debts accrued from having to pay bail, court and legal costs following arrest can indirectly increase vulnerability [4, 13, 26], as women agree to riskier clients or forms of sex during times of financial insecurity and hardship [27].

Although violence by intimate partners has been associated with increased HIV incidence among women in the general population [28], until recently there has been little exploration of the role that violence or arrest may play as a source of HIV risk among sex workers, using biological outcomes [29–32]. Nor has there been widespread consideration of whether integrating violence prevention into HIV programming could help reduce FSWs’ exposures to violence and arrests and hence their HIV risk [13, 26, 33].

Starting in 2003, the Karnataka Health Promotion Trust (KHPT), in partnership with the University of Manitoba, implemented a large-scale, comprehensive HIV prevention programme for more than 60,000 FSWs in Karnataka, south India. This was funded by the Bill and Melinda Gates Foundation, as part of the larger India AIDS Initiative (Avahan) programme [34]. At that time, HIV prevalence had reached over 30% among FSWs in some districts in the state. Although selling sex is not formally illegal in India (though profiting from sex work through brothels, pimping or trafficking is), many sex workers were unaware of their rights and sex workers and police were unclear about the law. The police could thus use threats of arrest to extort sex and otherwise harass them. The original Avahan model included outreach by peer educators, condom promotion and provision, and STI/HIV testing and treatment. Sex workers, however, reported that stigma and discrimination; violence by clients, pimps and the police; and police harassment and arrest were key concerns in their lives. In response, KHPT developed a complementary set of activities to address the structural drivers of FSW vulnerability, including violence and harassment, poverty and social inequity, and stigma and discrimination. Key components of the intervention included 1) the collectivization and mobilization of FSWs to enable critical thinking and to collectively fight injustices against them and campaign for their rights; 2) advocacy with and training of over 1880 policy makers, 1000 journalists and 12,700 police officers about the law, HIV and sex workers’ lives; 3) supporting the poorest FSWs to access state benefits and entitlements; and 4) a dedicated programme to prevent client and police violence. The violence prevention programme included the establishment of 24-hour crisis management teams in each district to support FSWs in the event of a violent attack. Moreover, it brought together a team of human rights lawyers who provided workshops for sex workers on their rights and helped bring perpetrators to justice. Further details on the intervention have been published previously [33, 35].

We have previously reported reductions in overall violence against FSWs (by any perpetrator) in Karnataka between 2005 and 2008, and associations between violence exposure (any perpetrator) and increased gonorrhoea prevalence [33]. In this paper, we extend this analysis to include data from 2005 to 2011, and we consider different forms of violence exposure (violence by non-partners and police arrest), as well as harassment and alcohol use. We examine 1) changes in reported violence and arrests over time and 2) associations between violence by non-partners (clients, police, pimps, strangers, rowdies) and police arrest and HIV/STI risk and prevalence. We use mediation analysis to explore the mechanisms underlying the associations found.

## Methods

### Integrated behavioural and biological assessments

A series of district-level, cross-sectional, integrated behavioural and biological assessment (IBBA) surveys were conducted with random samples of FSWs in four districts in Karnataka (Belgaum, Bellary, Shimoga and Bangalore), chosen purposively based on Karnataka's socio-cultural regions and the size of the high-risk population. Programmes were initiated in each district between April 2004 and October 2005, with Round 1 (R1) surveys conducted 10 to 16 months after programme initiation (August 2005 to July 2006), Round 2 (R2) surveys conducted 30 to 37 months after R1 (July 2008 to January 2009) and Round 3 (R3) surveys conducted 26 to 31 months after R2 (September 2010 to August 2011). Sample size calculations and sampling methodology have been described in detail previously [36, 37]. The target sample size per district was fixed at 400 completed interviews plus blood samples, except for Bangalore, where the sample size was enhanced to 800 to better represent the different sex work typologies (street vs. brothel vs. home-based) and the large number of FSWs in this city. A probability sampling method was used. Two different sampling approaches were adopted: 1) conventional cluster sampling was used for FSWs practising sex work at homes, brothels, lodges and *dhabas* (road-side eating establishments), where the population of FSWs was relatively stable; and 2) conventional time-location cluster (TLC) sampling (dividing a site into several TLCs and selecting the required number of TLCs randomly) was used for street-based FSWs.

A behavioural questionnaire was designed to be culturally sensitive and context specific, as previously described [36]. The questionnaire in all three survey rounds contained one question on sexual violence: *In the past one year, were you ever beaten or otherwise physically forced to have sexual intercourse with someone even though you didn't want to? (If yes) In the past one year, who was the person (or people) who physically forced you to have sexual intercourse against your will? Anyone else?* [38]. The questionnaires in R2 and R3 contained an additional question on physical violence: *In the last six months, how many times would you say someone has beaten you*? *(hurt, hit, slapped, pushed, kicked, punched, choked, burned?) (If* ≥*1) Who did this to you?*; one question on harassment: *Have you faced any problems and/or challenges that prevented you from safely practicing your work or accessing STI services in the last 6 months?* and one question on police arrest: *Have you ever been arrested? (If yes) How long ago were you last arrested? (less than a year ago/more than a year ago)*. Perpetrators of violence were defined as either “non-partners” (clients, strangers, police, goons, rowdies, auto-rickshaw drivers, pimps, madams) or “acquaintances” (relatives, neighbours, landlords, friends – usually of husbands/lovers, other sex workers). Unfortunately, the methodology for measuring intimate partner violence (IPV) changed between R2 and R3. For this reason, we have excluded violence by intimate partners from IBBA data analyses.

All interviews were conducted in the local language (Kannada) by trained interviewers. To ensure confidentiality, no identifying information was collected and data cannot be linked between rounds. Blood samples were taken to test for HIV and syphilis in all three survey rounds, and urine samples were collected to test for chlamydial infection and gonorrhoea in the first two rounds only, as previously described [36].

### Polling booth surveys

Polling booth surveys (PBS) are designed to minimize reporting bias, compared with face-to-face interview methods [39]. FSWs are separated from the interviewer and each other in a “polling booth” environment and asked a set of 23 questions derived from the IBBA, but with only yes/no answers, including one violence question: *In the past one year, were you ever beaten or otherwise physically forced to have sexual intercourse with someone even though you didn't want to?*; one question on condom use: *Did your last client use a condom with you?*; one question on self-efficacy of condom use, defined as answering “no” to: *During the past one month, was there a time when you wanted to use condom during sex but did not because your partner did not want to wear a condom?*; and one question on condom use when drinking: *During the past one month, was there a time when you intended to use a condom with a partner but did not use it because either of you had been drinking alcohol?*. Four rounds of PBS were conducted in 16 of the 20 KHPT districts between 2007 and 2011, including in the four IBBA districts. All FSWs who were contacted by the implementing non-governmental organization (NGO) or who received any service from the NGO (e.g. condom distribution, education outreach, collectivization, STI screening) were tracked using a computerized management information system (CMIS). All women tracked during the previous six months were included in the sampling frame. Women were stratified by location, sex worker typology and age to reflect the local distribution of FSWs according to these characteristics. Participants were randomly selected from the stratified lists and invited to participate in the PBS. Twenty women were invited to participate in each PBS and 12 to 21 PBS were conducted in each district at each survey round (proportional to the number of women who sold sex in that district); thus 240 to 410 women were invited to participate in PBS in each district at each time point. Demographic data were not collected in the PBS. The women sampled are unlikely to have differed substantially from those who participated in the IBBA, as all surveys were conducted in urban locations, sex work typologies are broadly similar across Karnataka and the stratification criteria used were the same for both surveys.

### Statistical analyses

All statistical analyses were performed in Stata version 12.0. For the IBBA data, survey analysis techniques were used to take account of the clustered nature of the data. Appropriate weights were used to account for the differential recruitment of FSWs by typology (street-based vs. brothel-based vs. home-based) within districts, differential non-response rates and differential probabilities of selection across districts.

**Figure 1:**
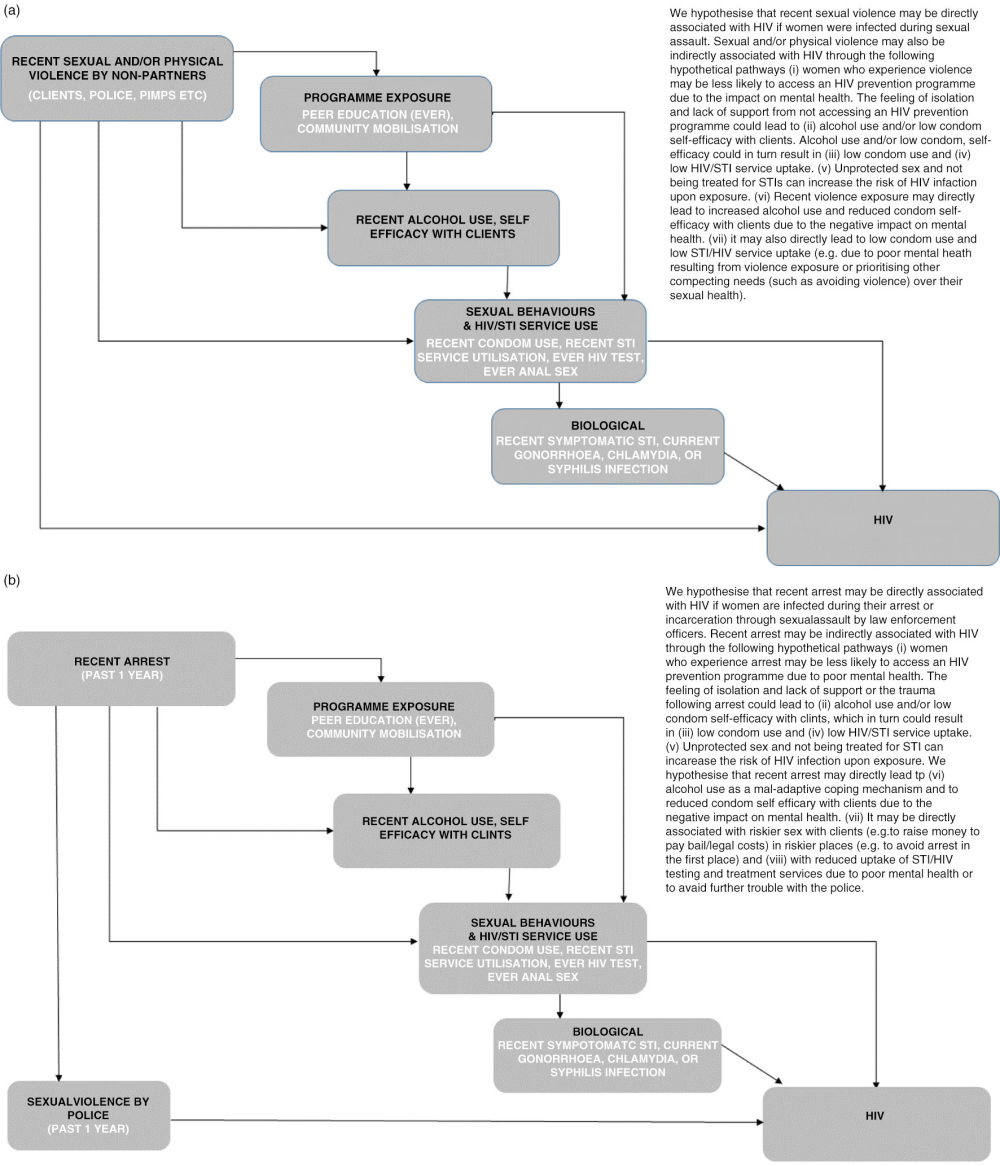
Simplified conceptual hierarchical framework for (a) violence and HIV infection, and (b) police arrest and HIV infection, among female sex workers in south India.

We first developed a conceptual model outlining our hypothesized pathways between violence/police arrest and HIV/STI risk and prevalence (Figures 1a and 1b). To examine temporal trends we used data from the PBS and IBBA surveys, with survey round as the exposure variable and violence, police arrest, harassment, alcohol use and condom self-efficacy with clients as outcome variables. We performed descriptive and multivariate logistic regression analyses to examine associations over time. For the PBS analyses we examined temporal trends in violence by any partner, whereas for the IBBA analyses we examined temporal trends in violence by non-partners. We next examined associations between violence by non-partners/police arrest and HIV/STI risk and prevalence, using IBBA data from R2 and R3 (where both violence questions were asked). We used descriptive and multivariate analyses to examine associations between violence or arrests and 1) exposure to the HIV prevention programme; 2) alcohol use in the past week and condom self-efficacy with clients; 3) sexual behaviour and uptake of HIV/STI services; and 4) HIV and STI infection.

Odds ratios (ORs) were used as the measure of association, and the Wald chi-square test was the statistical test employed. The adjusted Wald test was used to test for effect modification. Multivariate analyses, adjusting for district, survey round, sociodemographic and sex work characteristic confounders, were performed for IBBA data only, as the PBS methodology precludes the collection of linked data.

To explore whether intermediary variables were mediators on the pathway between violence by non-partners or police arrest and HIV prevalence (Figures 1a and 1b), we conducted mediation analyses [40]. Potential mediating factors associated with both the exposure (violence or arrest) and the outcome (HIV infection) variables were added to the adjusted model. Factors that shifted the adjusted OR (AOR) towards 1.0 were deemed to mediate the effect between violence/arrests and HIV infection. For a full mediating effect, the association between violence/arrests and HIV prevalence would become non-significant once the mediating factor(s) was added to the adjusted model. For a partial mediating effect, the AOR would shift closer to 1.0, but remain significant.

### Ethical considerations

Statutory approval for the conduct of the IBBAs and their protocols was obtained from the Government of India's Health Ministry Screening Committee. All studies were approved by the Institutional Ethical Review Board of St. John's Medical College, Bangalore, India, and the Research Ethics Board of the University of Manitoba, Winnipeg, Canada.

## Results

### Study population

Five thousand seven hundred ninety-two FSWs participated in the IBBAs (R1: 1883; R2: 1975; R3: 1934) and 15,813 FSWs participated in the PBS (R1: 3631; R2: 3953; R3: 3939; R4: 4290). From all PBS rounds, 19.4% of women reported being raped by *anyone* in the past year, and from all IBBA rounds, 7.6% of women reported being raped by a *non-partner* in the past year. In IBBA R2 and R3, 6.9% reported being beaten in the past six months by a *non-partner* and 6.5% reported being raped in the past year by a *non-partner*; 11.1% reported being raped in the past year and/or beaten in the past six months by a *non-partner*. The most common “non-partner” perpetrators were clients (5.9% raped in the past year; 4.4% beaten in the past six months) and strangers (0.4 and 2.0%, respectively), with <1% reporting either type of violence by police, pimps, madams or “rowdies.”

### Temporal changes in violence, harassment, police arrest, alcohol use and condom self-efficacy

We first examined temporal changes in reported violence, harassment, arrests, alcohol use and condom self-efficacy, using two data sources (PBS and IBBA).

#### Polling booth surveys

**Figure 2:**
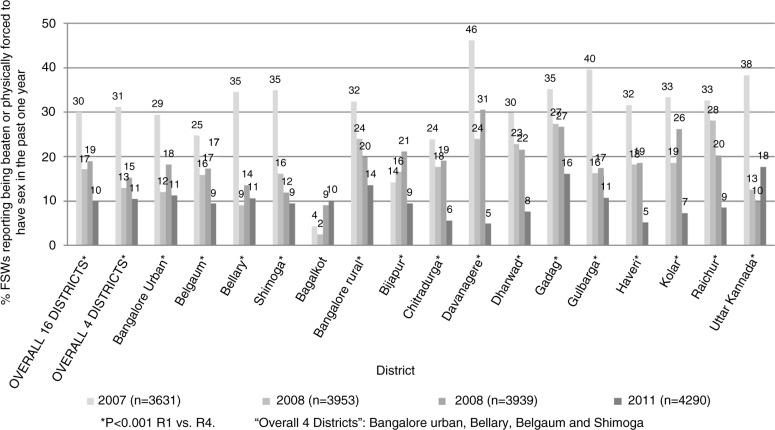
Reductions in reported violence over time in 16 districts in Karnataka (2007 to 2011): polling booth surveys.

In 2007, data from 3631 women across 16 districts showed that 30.0% of participants reported being raped in the past one year. In addition, 69.8% reported using a condom with their last client, 60.8% reported self-efficacy to use a condom and 34.5% reported not using a condom in the past month because she or her partner had been drinking alcohol. When we examined changes over time between 2007 and 2011, we found a significant stepwise reduction in the proportion of women who reported being raped in the past year (30.0% in 2007, 17.1% in 2008, 19.0% in 2009, 10.0% in 2011, *p<*0.001) (Figure 2). Indeed, in every district surveyed except two (Bagalkot and Bijapur), by the final survey round, the proportion of women who reported being raped in the past one year was significantly lower (*p<*0.001) compared with baseline (Figure 2). In addition, we found a significant increase over time in the proportion who reported using a condom with their last client (69.8% in 2007, 74.5% in 2008, 71.8% in 2009, 81.8% in 2011, *p<*0.001) and in the proportion reporting condom self-efficacy (60.8% in 2007, 70.0% in 2008, 66.9% in 2009, 74.0% in 2011, *p<*0.001), as well as a significant reduction over time in the proportion reporting not using a condom in the past month because she or her partner had been drinking alcohol (34.5% in 2007, 21.5% in 2008, 27.1% in 2009, 19.6% in 2011, *p<*0.001). In the four districts where the IBBA surveys were conducted, similar findings were observed (Supplementary Table 1).

#### Integrated behavioural and biological assessments

When we examined temporal changes using IBBA data [R1 (2005); R2 (2008); R3 (2011)] in univariate and adjusted analyses, the proportion of women who reported being raped by a non-partner in the past one year fell significantly between R1 and R2 (9.8% vs. 5.2%), and there was weak evidence of a reduction between R1 and R3 (9.8% vs. 7.8%; Table 1). In addition, between R2 and R3, there was a significant reduction in the proportion of women who reported being beaten by a non-partner in the past six months (8.4% vs. 5.5%). When we examined data for clients only, the results followed a similar trend (Table 1). We also found significant reductions in the proportion of FSWs reporting harassment in the past six months (10.9% vs. 6.6%) and in the proportion reporting being arrested in the past one year (5.5% vs. 3.0%), as well as a significant stepwise reduction over time in the proportion of women reporting alcohol use in the past one week (R1: 32.5%; R2: 24.8%; R3: 16.9%). There were no changes in the proportion of women reporting condom-related self-efficacy (Table 1).

**Table 1:** Temporal changes in violence, harassment, arrests, alcohol use and condom self-efficacy in four districts (IBBA)

			Round 1 (*n=*1883)	Round 2 (*n=*1975)	Round 3 (*n=*1934)
Beaten or otherwise physically forced to have sex in the past one year					
Non-partner	Client, stranger, pimp, police, rowdy, auto driver	% Crude OR Adjusted OR	9.8 Ref Ref	5.2 0.51 (0.36, 0.70)*** 0.56 (0.40, 0.80)**	7.8 0.78 (0.59, 1.02) *p=*0.075 0.80 (0.60, 1.07) *p=*0.13
	Client	% Crude OR Adjusted OR	7.3 Ref Ref	3.9 0.52 (0.35, 0.76)** 0.58 (0.39, 0.87)**	6.6 0.90 (0.68, 1.21) 0.90 (0.66, 1.25)
Acquaintance	Relative, neighbour, landlord, friend (of husband)	% Crude OR Adjusted OR	0.7 Ref Ref	0.7 1.05 (0.43, 2.57) 1.04 (0.23, 2.65)	0.5 0.65 (0.25, 1.69) 0.70 (0.23, 2.14)
Beaten (hurt, hit, slapped, pushed, kicked, punched, choked, burned) in the past six months					
Non-partner	Client, stranger, pimp, police, rowdy, auto driver	% Crude OR Adjusted OR	–	8.4 Ref Ref	5.5 0.63 (0.46, 0.87)** 0.69 (0.49, 0.95)*
	Client	% Crude OR Adjusted OR	–	5.0 Ref Ref	3.8 0.74 (0.53, 1.05) *p=*0.095 0.80 (0.56, 1.13)
Acquaintance	Relative, neighbour, landlord, friend (of husband)	% Crude OR Adjusted OR	–	1.7 Ref Ref	1.8 1.09 (0.58, 2.01) 1.17 (0.63, 2.19)
Faced any problems and/or challenges that prevented safely practicing sex work or accessing STI services in the past six months					
(harassment)		% Crude OR Adjusted OR	–	10.9 Ref Ref	6.6 0.57 (0.42, 0.78)*** 0.53 (0.38, 0.75)***
Arrested in the past one year		% Crude OR Adjusted OR	–	5.5 Ref Ref	3.0 0.54 (0.34, 0.85)** 0.57 (0.35, 0.93)*
Alcohol use in the past week		% Crude OR Adjusted OR	32.5 Ref Ref	24.8 0.69 (0.56, 0.85)** 0.71 (0.58, 0.87)**	16.9 0.42 (0.34, 0.53)*** 0.44 (0.35, 0.56)***
Answering *no* to *In past month had time when wanted to use a condom with a client but did not* (condom *self-efficacy*) %		% Crude OR Adjusted OR	-	86.2 Ref Ref	87.2 1.09 (0.87, 1.36) 1.26 (0.98, 1.61)

* *p<*0.05;

** *p<*0.01;

*** *p* <0.0001. IBBA, integrated behavioural and biological assessment; OR, odds ratio. Data from four districts (Bangalore, Belgaum, Bellary, Shimoga). Models adjusted for age, district, sex work typology (place where sex worker solicits clients), marital status and sold sex outside of the district (migrant sex worker).

### Violence by non-partners and associations with HIV/STI risk and prevalence

We next examined whether there were associations between reported exposures to violence by non-partners and HIV/STI risk and prevalence. We conducted cross-sectional analyses using pooled data from IBBA R2 and R3, where both violence questions were asked. Overall, 6.5% of women reported being raped by a non-partner in the past one year, 6.9% reported being beaten by a non-partner in the past six months, 11.1% reported at least one of the violence exposures and 2.3% reported both violence exposures.

Women who reported being raped in the past one year and/or beaten in the past six months by a non-partner were likely to be younger (<30 years old), to not have an additional source of income to sex work and to be previously married but now widowed, deserted or divorced and living alone. They were likely to report starting sex work at a younger age; to have sex with clients in brothels, lodges or *dhabas* (rather than at home or on the streets); to entertain more clients per week; to be a migrant sex worker; and to have previously sold sex in Mumbai (Table 2).

**Table 2:** Sociodemographic and sex work characteristics of female sex workers, and violence by non-partner(s) and arrests in the past one year: pooled data from four districts, IBBA R2 and R3

	Violence by non-partner(s)^a^				Arrested in the past one year		
		
Characteristic	No (*n=*3493)	Either (*n=*325)	Both (*n=*91)	*p*-value χ^2^ test^b^	No (*n=*3748)	Yes (*n=*157)	*p*-value χ^2^ test
**Current age (years)**							
<25 25 to 29 30 to 39 40+ Mean	15.6 20.3 42.4 21.7 32.70	17.6 26.2 41.1 15.2 31.25	24.0 28.8 32.0 15.3 30.06	0.0099	15.4 21.2 41.9 21.4 32.61	27.7 16.8 44.5 10.9 30.25	0.0008
**Literacy**							
** **Literate	35.9	37.1	36.5	0.93	36.3	28.4	0.12
**Additional income to sex work**							
** **Yes	65.7	54.5	55.0	0.0005	65.4	43.8	<0.001
**Marital status**							
** **Never married/*devadasi* Married Widowed/divorced/deserted (live with partner) Widowed/divorced/deserted (live alone)	17.8 37.0 10.6 34.7	18.8 29.1 10.5 41.6	19.1 22.7 8.9 49.3	0.035	17.8 36.3 10.4 35.5	20.6 27.7 13.0 38.7	0.24
**Regular partner**							
** **Yes	63.3	60.2	64.4	0.62	63.4	55.5	0.11
**Children**							
** **0 ** **1 to 2 ** **3+ ** **Mean	12.0 56.4 31.6 1.60	17.5 53.3 29.1 1.49	23.8 61.7 14.5 1.36	0.0007	12.4 56.2 31.5 1.59	21.4 57.3 21.3 1.41	0.0037
**Local dweller**							
** **Living in birth district	79.3	76.3	68.4	0.058	79.4	66.7	0.0003
**District**							
** **Belgaum ** **Bellary ** **Shimoga ** **Bangalore	23.2 24.3 25.2 27.3	29.8 25.9 22.1 22.3	30.9 20.1 18.9 30.2	0.085	23.5 24.3 25.4 26.8	34.2 25.3 10.8 29.7	0.010
**IBBA round**							
** **2 ** **3	50.4 49.6	53.7 46.3	46.2 53.8	0.46	49.9 50.1	64.9 35.2	0.0067
**Age at sexual debut (years)**							
<14 ** **Mean	21.9 15.63	26.0 15.34	31.6 15.32	0.071	22.2 15.62	27.2 15.16	0.18
**Age started sex work (years**)							
** **<20 ** **20 to 24 ** **25 to 29 ** **30+ ** **Mean	19.8 24.5 24.5 31.2 25.88	29.6 26.3 24.2 20.0 23.69	27.6 31.3 16.1 25.1 23.72	0.0001	20.4 24.8 24.4 30.5 25.73	31.4 26.4 20.9 21.2 23.49	0.0049
**Duration of sex work (years)**							
** **0 to 1 ** **2 to 4 ** **5 to 9 ** **10+ ** **Mean	17.4 32.8 24.0 25.9 6.82	15.3 30.5 25.9 28.3 7.56	30.0 25.3 14.9 29.8 6.34	0.056	17.3 32.5 24.1 26.2 6.88	21.5 30.0 21.4 27.0 6.76	0.62
**Usual place of solicitation**							
** **Home ** **Brothel/lodge/*dhaba* ** **Public places ** **Phone	29.0 6.9 42.4 21.8	20.8 13.1 47.9 18.1	14.1 12.9 47.5 25.6	0.0001	28.5 7.2 42.3 22.0	15.7 15.4 57.6 11.3	<0.001
**Usual place of having sex with clients**							
** **Home ** **Brothel/lodge/*dhaba* ** **Public places	64.1 24.7 11.2	48.4 38.3 13.4	42.1 45.3 12.6	<0.001	63.7 25.1 11.2	30.1 54.7 15.2	<0.001
**Clients per typical week**							
** **1 to 5 ** **6 to 9 ** **10+ ** **Mean	39.4 27.7 32.9 9.24	23.5 26.2 50.3 14.86	20.8 24.9 54.4 15.50	<0.001	38.5 27.7 33.9 9.52	18.0 24.4 57.6 17.99	<0.001
**Charge for sex with last client**							
** **>250 rupees ** **Mean (rupees)	46.8 334.73	43.3 296.78	38.1 311.40	0.23	46.6 332.26	39.4 299.48	0.18
**Income typical week sex work**							
** **>2000 rupees ** **Mean (rupees)	37.5 2837.86	49.6 3481.31	52.9 4307.57	<0.001	38.1 2860.31	57.6 4456.91	<0.001
**Ever sex work outside district**							
** **Migrant	11.84	20.85	36.29	<0.001	12.4	30.7	<0.001
**Sex work in Mumbai ever**							
** **Yes	2.03	5.00	7.36	0.0001	2.1	8.7	<0.001

IBBA, integrated behavioural and biological assessment; R2, Round 2; R2, Round 3. Data from IBBA R2 and R3, four districts (Bangalore, Belgaum, Bellary, Shimoga).

a Violence by non-partner(s) defined as “beaten or otherwise physically forced to have sexual intercourse in past one year *and/or* beaten in the past six months by non-partner”;

b χ^2^ test comparing “none” vs. “either” vs. “both.”

When we examined exposure to recent physical violence, sexual violence or either physical or sexual violence by a non-partner, the results were very similar (Supplementary Tables 2 and 3). We therefore present results for women who reported either violence type (Table 3). In both univariate and adjusted analyses, women who reported being raped in the past one year and/or beaten in the past six months by a non-partner reported similar exposure to the HIV prevention programme and to community mobilization activities and had similar uptake of HIV and STI clinical services (Table 3). However, exposure to violence by non-partners was significantly associated with increased HIV prevalence among FSWs. It was also associated with alcohol use in the past week, reduced condom self-efficacy with clients, anal sex ever with a client, reduced condom use with last client, reduced condom use with last repeat client and increased STI prevalence [symptomatic STI during the past year, gonorrhoea infection and syphilis infection (Table 3)].

**Table 3:** Violence by non-partners and arrests in the past year and associations with HIV/STI risk and prevalence (pooled data from four districts, IBBA rounds 2 and 3)

	Violence by non-partner(s)^a^			Arrested in the past one year		
		
	No (*n=*3493)	Yes (*n=*416)	*p*-value Wald test	No (*n=*3748)	Yes (*n=*161)	*p*-value Wald test
**Programme exposure**						
Ever seen a peer educator (%)	96.4	95.8		96.3	97.7	
Crude OR	0.85 (0.46, 1.59)		0.61	1.62 (0.60, 4.38)		0.34
Adjusted OR	0.82 (0.47, 1.45)		0.50	2.43 (0.83, 7.12)		0.10
Ever seen a condom demonstration (%)	91.1	93.0		91.2	94.0	
Crude OR	1.31 (0.83, 2.07)		0.25	1.51 (0.78, 2.93)		0.22
Adjusted OR	1.19 (0.76, 1.87)		0.44	1.72 (0.84, 3.54)		0.14
Ever been to an NGO meeting (%)	84.9	84.2		84.9	83.1	
Crude OR	0.95 (0.70, 1.30)		0.77	0.87 (0.56, 1.37)		0.55
Adjusted OR	1.05 (0.77, 1.43)		0.76	1.23 (0.78, 1.94)		0.37
Ever been to a drop-in centre (%)	67.3	67.4		66.9	77.7	
Crude OR	1.00 (0.79, 1.27)		0.99	1.73 (1.12, 2.66)		0.013
Adjusted OR	0.96 (0.75, 1.22)		0.74	1.51 (0.95, 2.42)		0.084
Member of a FSW collective or peer group (%)	51.9	50.1		51.4	58.7	
Crude OR	0.93 (0.74, 1.18)		0.55	1.34 (0.88, 2.04)		0.17
Adjusted OR	0.94 (0.73, 1.20)		0.62	1.46 (0.95, 2.23)		0.084
**Alcohol use and self-efficacy**						
Alcohol use past one week (%)	19.1	35.0		19.7	48.1	
Crude OR	2.27 (1.77, 2.92)		<0.001	3.79 (2.61, 5.49)		<0.001
Adjusted OR	1.98 (1.54, 2.53)		<0.001	2.79 (1.93, 4.04)		<0.001
Answering *no* to *In past month had time when wanted to use a condom with a client but did not* (condom *self-efficacy*) %	88.3	73.7		87.0	80.3	
Crude OR	0.37 (0.28, 0.48)		<0.001	0.61 (0.39, 0.97)		0.035
Adjusted OR	0.36 (0.27, 0.47)		<0.001	0.62 (0.39, 0.98)		0.039
**Sexual behaviour and HIV/STI service uptake**						
Ever had anal sex with client (%)	11.7	27.9		13.2	18.6	
Crude OR	2.94 (2.22, 3.90)		<0.001	1.50 (0.92, 2.45)		0.11
Adjusted OR	2.98 (2.22, 4.00)		<0.001	1.50 (0.92, 2.45)		0.10
Condom use last sex client	92.01	85.7		91.47	87.73	
Crude OR	0.52 (0.38, 0.72)		<0.001	0.67 (0.39, 1.15)		0.14
Adjusted OR	0.47 (0.34, 0.64)		<0.001	0.57 (0.33, 1.01)		0.054
Condom use last sex occasional client^b^ (%)	93.9	92.8		93.8	92.8	
Crude OR	0.84 (0.55, 1.28)		0.42	0.85 (0.44, 1.65)		0.63
Adjusted OR	0.70 (0.46, 1.06)		0.095	0.73 (0.37, 1.46)		0.38
Condom use last sex repeat client^c^ (%)	90.3	84.6		89.6	89.9	
Crude OR	0.59 (0.43, 0.82)		0.002	1.03 (0.56, 1.89)		0.92
Adjusted OR	0.55 (0.39, 0.76)		<0.001	1.06 (0.57, 1.95)		0.86
Condom use last sex regular partner^d^ (%)	36.4	38.5		36.5	39.9	
Crude OR	1.10 (0.83, 1.44)		0.52	1.16 (0.77,1.75)		0.49
Adjusted OR	0.75 (0.54, 1.05)		0.095	1.01 (0.61, 1.69)		0.96
Condom use last anal sex client (%)	78.8	68.9		77.4	63.5	
Crude OR	0.60 (0.36, 0.99)		0.044	0.51 (0.23, 1.10)		0.087
Adjusted OR	0.64 (0.37, 1.08)		0.096	0.80 (0.31, 2.05)		0.64
STI clinic past six months (%)	80.6	83.3		80.7	86.3	
Crude OR	1.20 (0.88, 1.65)		0.25	1.50 (0.89, 2.53)		0.12
Adjusted OR	1.20 (0.88, 1.64)		0.25	1.74 (1.02, 2.98)		0.042
HIV test ever (%)	79.2	79.5		79.3	77.4	
Crude OR	1.02 (0.78, 1.33)		0.87	0.89 (0.54, 1.47)		0.66
Adjusted OR	1.09 (0.81, 1.48)		0.56	1.22 (0.74, 2.03)		0.44
**HIV and STI infection**						
HIV (%)	11.8	20.0		12.2	23.8	
Crude OR	1.87 (1.40, 2.49)		<0.001	2.26 (1.46, 3.48)		<0.001
Adjusted OR	1.59 (1.18, 2.15)		0.002	1.91 (1.17, 3.12)		0.010
Symptomatic STI past 12 months^e^ (%)	34.8	59.2		36.6	57.1	
Crude OR	2.72 (2.17, 3.41)		<0.001	2.30 (1.62, 3.25)		<0.001
Adjusted OR	2.62 (2.07, 3.30)		<0.001	2.17 (1.51, 3.13)		<0.001
Chlamydia^f^ (%)	5.8	6.4		5.5	12.4	
Crude OR	1.11 (0.58, 2.13)		0.75	2.42 (1.27, 4.60)		0.007
Adjusted OR	1.07 (0.54, 2.12)		0.85	1.80 (0.92, 3.53)		0.087
Gonorrhoea^f^ (%)	2.0	6.5		2.1	8.9	
Crude OR	3.46 (1.96, 6.13)		<0.001	4.51 (1.83, 11.09)		0.001
Adjusted OR	2.79 (1.51, 5.15)		0.001	2.69 (0.96, 7.56)		0.060
Reactive syphilis (%)	5.9	9.0		5.8	16.2	
Crude OR	1.56 (1.04, 2.35)		0.033	3.12 (1.93, 5.06)		<0.001
Adjusted OR	1.40 (0.91, 2.14)		0.12	2.63 (1.54, 4.15)		<0.001
High-titre syphilis (%)	2.3	4.5		2.3	8.8	
Crude OR	2.02 (1.09, 3.74)		0.026	4.13 (2.18, 7.81)		<0.001
Adjusted OR	1.86 (1.04, 3.31)		0.036	3.35 (1.78, 6.28)		<0.001

IBBA, integrated behavioural and biological assessment; OR, odds ratio. Data from IBBA Rounds 2 and 3, four districts (Bangalore, Belgaum, Bellary, Shimoga). Models adjusted for district, IBBA round, place where sex worker has sex with clients, age, marital status, additional source of income to sex work and ever sold sex outside the district (migrant sex worker).

a Violence by non-partner(s) defined as “beaten or otherwise physically forced to have sexual intercourse in past one year *and/or* beaten in the past six months, by non-partner”;

b occasional client defined as “client who has come to you only once or a few times but you do not remember their face or do not know them”;

c repeat client defined as “client you recognize well, who has come to you repeatedly and you know them”;

d regular partner defined as “a main (regular) male sexual partner who does not pay to have sex with you”;

e sypmtomatic STI past 12 months defined as vaginal discharge, lower abdominal pain (not diarrhoea/menses) and/or genital ulcer/sore;

f data available for Round 2 only.

### Police arrest in the past year and associations with HIV/STI risk and prevalence

We next examined associations between being arrested in the past year and HIV/STI risk and infection. Women who reported being arrested in the past one year had similar sociodemographic and sex work characteristics to those who reported violence by a non-partner (Table 2). Interestingly, in both univariate and multivariate analyses, we again found strong associations between being arrested in the past one year and HIV prevalence (Table 3). Being arrested in the past year was also associated with alcohol use in the past week, reduced condom self-efficacy with clients, reduced condom use at last sex with a client, increased uptake of STI services and STI prevalence (increased reporting of a symptomatic STI during the past year; gonorrhoea infection; or syphilis infection) (Table 3). There were borderline associations between police arrest and several programme exposure variables (ever seen a peer educator, ever seen a condom demonstration, ever visited a drop-in centre and membership in a peer group or FSW collective) (Table 3).

### Mediation analyses

We next conducted mediation analyses. We first examined if potential mediators were associated with the exposure variable (violence or arrest) (Table 3) and with the outcome variable (HIV infection) (Table 4). Alcohol use in the past week, anal sex ever with a client, symptomatic STI in the past one year, gonorrhoea infection, reactive syphilis infection and high-titre syphilis infection were associated (*p<*0.05) with violence/arrests *and* with HIV infection. In a stepwise fashion, we added each potential mediator to the final adjusted model, although we could not include gonorrhoea or chlamydia as data were only available from R2 of the survey (Table 5). The addition of alcohol use in the past week, symptomatic STI in the past one year and reactive syphilis infection all shifted the AOR closer to 1.0 (Table 5). When all three of these variables were added to the model with violence by non-partners as the explanatory variable, the AOR shifted from 1.59 (1.18, 2.15) to 1.44 (1.06, 1.96), with a proportional reduction in the AOR of 9.4%. Similarly, when all three of these variables were added to the model with police arrest as the explanatory variable, the AOR shifted from 1.91 (1.17, 3.12) to 1.62 (1.00, 2.63), with a proportional reduction in the AOR of 15.2% (Table 5). These analyses suggest that alcohol use in the past week, symptomatic STI in the past one year and reactive syphilis infection may be partially mediating the effect between violence by non-partners/police arrest and HIV infection.

**Table 4:** Analysis of the association between factors on the conceptual pathway between violence by non-partners/police arrest and HIV infection among female sex workers in northern Karnataka (pooled data from four districts, IBBA Rounds 2 and 3)

	HIV infection			
			
Explanatory variable	No *n=*3493 (%)	Yes *n=*416 (%)	Crude odds ratio	*p*-value Wald test
Violence by non-partner (raped in the past year/beaten past six months)^a^	10.2	17.6	1.87 (1.40, 2.49)	< 0.001
Police arrest past year	3.7	8.0	2.26 (1.46, 3.48)	< 0.001
*Programme exposure*						
1. Ever seen a peer educator	96.2	97.3	1.44 (0.84, 2.47)	0.18
2. Ever seen a condom demonstration	90.9	94.0	1.55 (1.05, 2.29)	0.03
3. Ever been to an NGO meeting	84.6	86.4	1.51 (1.18, 1.92)	0.001
4. Ever been to a drop-in centre	66.2	74.7	1.15 (0.84, 1.58)	0.37
5. Member of an FSW collective or peer group	51.4	53.0	1.06 (0.85, 1.33)	0.59
*Alcohol use and condom self-efficacy*						
1. Alcohol use past week	19.9	27.7	1.54 (1.21, 1.97)	< 0.001
2. Condom self-efficacy with clients	86.6	87.0	1.04 (0.76, 1.42)	0.82
*Condom use and sexual behaviour*						
1. Ever had anal sex with client	14.0	10.1	0.69 (0.47, 1.00)	0.05
2. Condom use last sex client	91.0	93.2	1.36 (0.92, 1.99)	0.12
3. Condom use last sex occasional client^b^	93.5	95.1	1.35 (0.85, 2.14)	0.21
4. Condom use last sex repeat client^c^	89.3	91.3	1.25 (0.83, 1.88)	0.28
5. Condom use during last sex with regular partner^d^	34.4	57.7	2.60 (1.88, 3.60)	< 0.001
6. Condom use during last anal sex with client (*n*=497)	77.7	64.9	0.53 (0.24, 1.19)	0.12
*HIV/STI clinic use*						
1. STI clinic in the past six months	80.6	83.3	1.21 (0.87, 1.66)	0.25
2. HIV test ever	79.2	80.2	1.06 (0.83, 1.36)	0.62
*STI infection*						
1. Symptomatic STI past 12 months^e^	36.9	42.7	1.28 (1.01, 1.61)	0.040
2. Chlamydia^f^ (*n*=1952)	6.0	5.2	0.85 (0.45, 1.63)	0.63
3. Gonorrhoea^f^ (*n*=1952)	2.2	4.3	2.03 (1.14, 3.62)	0.016
4. Reactive syphilis	5.2	13.8	2.94 (2.14, 4.04)	< 0.001
5. High-titre syphilis	2.2	4.8	2.20 (1.19, 4.05)	0.012

IBBA, integrated behavioural and biological assessment; NGO, non-governmental organization; FSW, female sex worker. Data from IBBA Rounds 2 and 3, four districts (Bangalore, Belgaum, Bellary, Shimoga).

a Violence by non-partner(s) defined as “beaten or otherwise physically forced to have sexual intercourse in past one year *and/or* beaten in the past six months by a non-partner”; non-partner defined as “client, stranger, pimp, police, rowdy, auto driver”;

b occasional client defined as “client who has come to you only once or a few times but you do not remember their face or do not know them”;

c repeat client defined as “client you recognize well, who has come to you repeatedly and you know them”;

d regular partner defined as “a main (regular) male sexual partner who does not pay to have sex with you”;

e symptomatic STI past 12 months defined as vaginal discharge, lower abdominal pain (not diarrhoea/menses) and/or genital ulcer/sore (past 12 months);

f gonorrhoea and chlamydia infection were measured in IBBA Round 2 only.

**Table 5:** Analysis of the effect of mediating factors on the association between HIV prevalence and 1) violence by non-partners and 2) arrests in the past one year, among female sex workers in northern Karnataka (pooled data from four districts, IBBA Rounds 2 and 3)

Explanatory variable	Crude odds ratio	*p-*value Wald test	Factors adjusted for in model	Adjusted odds ratio	*p-*value Wald test	Proportional difference in AOR^a^
Violence by non-partner^b^ (raped in the past year/beaten in the past six months)	1.87 (1.40, 2.49)	<0.001	Sociodemographic variables^c^		1.59 (1.18, 2.15)	0.003	
			*Programme exposure*				
			1. Sociodemographic variables+ever seen a peer educator		1.60 (1.18, 2.16)	0.002	
			2. Sociodemographic variables+ever seen a condom demonstration		1.59 (1.17, 2.15)	0.003	
			3. Sociodemographic variables+ever been to an NGO meeting		1.59 (.181, 2.15)	0.003	
			4. Sociodemographic variables+ever been to a drop-in centre		1.59 (1.18, 2.15)	0.002	
			5. Sociodemographic variables+member of an FSW collective/peer group		1.59 (1.18, 2.15)	0.002	
			*Alcohol use and condom self-efficacy*				
			1. Sociodemographic variables+alcohol use past week		**1.54 (1.13, 2.09)**	**0.006**	**3.1%**
			2. Sociodemographic variables+condom self-efficacy with clients		1.57 (1.16, 2.11)	0.003	
			*Condom use and sexual behaviour*				
			1. Sociodemographic variables+ever had anal sex with client		1.69 (1.24, 2.30)	0.001	
			2. Sociodemographic variables+condom use last sex client		1.61 (1.19, 2.18)	0.002	
			*HIV/STI clinic use*				
			1. Sociodemographic variables+STI clinic past six months		1.59 (1.18, 2.15)	0.002	
			2. Sociodemographic variables+HIV test ever		1.58 (1.17, 2.14)	0.003	
			*STI infection*				
			1. Sociodemographic variables+symptomatic STI past one year		**1.53 (1.13, 2.07)**	**0.006**	**3.8%**
			2. Sociodemographic variables+reactive syphilis		**1.55 (1.15, 2.10)**	**0.004**	**2.5%**
			3. Sociodemographic variables+reactive syphilis+symptomatic STI past 12 months		**1.48 (1.09, 2.00)**	**0.011**	**6.9%**
			***Final model***				
			***Sociodemographicvariables+reactive syphilis+symptomatic STI past 12 months+alcohol use past week***		**1.44 (1.06, 1.96)**	**0.021**	**9.4%**
Police arrest in the past year	2.26 (1.46, 3.48)	<0.001	Sociodemographic variables^c^		1.91 (1.17, 3.12)	0.010	
			*Programme exposure*				
			1. Sociodemographic variables+ever seen a peer educator		1.90 (1.17, 3.11)	0.010	
			2. Sociodemographic variables+ever seen a condom demonstration		1.90 (1.16, 3.09)	0.010	
			3. Sociodemographic variables+ever been to an NGO meeting		1.91 (1.17, 3.11	0.010	
			4. Sociodemographic variables+ever been to a drop-in centre		1.90 (1.17, 3.08)	0.010	
			5. Sociodemographic variables+member of an FSW collective/peer group		1.92 (1.17, 3.13)	0.009	
			*Alcohol use and condom self-efficacy*				
			1. Sociodemographic variables+alcohol use past week		**1.80 (1.09, 2.97)**	**0.021**	**5.8%**
			2. Sociodemographic variables+condom self-efficacy with clients		1.92 (1.18, 3.12)	0.009	
			*Condom use and sexual behaviour*				
			1. Sociodemographic variables+ever had anal sex with client		1.95 (1.19, 3.17)	0.008	
			2. Sociodemographic variables+condom use last sex client		1.92 (1.18, 3.14)	0.009	
			*HIV/STI clinic use*				
			1. Sociodemographic variables+STI clinic past six months		1.90 (1.16, 3.11)	0.010	
			2. Sociodemographic variables+HIV test ever		1.91 (1.18, 3.09)	0.009	
			*STI infection*				
			1. Sociodemographic variables+symptomatic STI past 12 months^d^		**1.84 (1.13, 3.00)**	**0.014**	**3.7%**
			2. Sociodemographic variables+reactive syphilis		**1.76 (1.09, 2.84)**	**0.020**	**7.9%**
			3. Sociodemographic variables+reactive syphilis+symptomatic STI past 12 months		**1.69 (1.05, 2.72)**	**0.029**	**11.5%**
			***Final model***				
			**Sociodemographic variables+reactive syphilis+symptomatic STI past 12 months+alcohol use past week**		**1.62 (1.00, 2.63)**	**0.050**	**15.2%**

IBBA, integrated behavioural and biological assessment; NGO, non-governmental organization; FSW, female sex worker; OR, odds ratio; AOR, adjusted odds ratio. Data from IBBA Rounds 2 and 3, four districts (Bangalore, Belgaum, Bellary, Shimoga). Gonorrhoea and chlamydia infection were measured in IBBA Round 2 only and therefore could not be included as potential mediating factors in these models. Bold represents a shift in the Adjusted Odds Ratio towards 1.0.

a Proportional difference in AOR calculated for models where the addition of mediating variable(s) to the model adjusted for sociodemographic confounders shifted the AOR towards 1.0: [OR adjusted for sociodemographic confounders – OR adjusted for sociodemographic confounders and mediating variables]/OR adjusted for sociodemographic confounders);

b violence by non-partner(s) defined as “beaten or otherwise physically forced to have sexual intercourse in the past year *and/or* beaten in the past six months by a non-partner”; non-partner defined as “client, stranger, pimp, police, rowdy, auto driver”;

c models adjusted for district, IBBA round, place where sex worker has sex with clients, age, marital status, additional source of income to sex work and ever sold sex outside the district (migrant sex worker);

d symptomatic STI past 12 months defined as “vaginal discharge, lower abdominal pain (not diarrhoea/menses) and/or genital ulcer/sore (during the past 12 months).”

## Discussion

Our findings suggest that in the KHPT HIV prevention programme districts, alongside declines in HIV prevalence [41], there were significant reductions in the proportion of FSWs reporting sexual violence, harassment, arrests and violence by non-partners between 2005 and 2011. There were also significant reductions over time in reported alcohol use. These trends were observed both through PBS across 16 districts and through in-depth IBBAs across four districts. This extends our previous findings and, to our knowledge, is the first study to report such changes, following a large-scale HIV and violence prevention programme.

Previously (using data from 2005 to 2008) we reported strong associations between violence (*by any perpetrator*) and gonorrhoea prevalence, but we did not find associations between violence and HIV or syphilis infection [33]. In the current study (using data from 2008 to 2011), we considered different forms of violence exposure and found strong associations between violence by non-partners and police arrest and increased HIV, syphilis and gonorrhoea prevalence. Although other studies have reported associations between violence or arrest and proximate determinants of risk (such as reduced condom use) [4, 8, 42–44], this study is among the first to report associations between violence exposure or police arrest and biological outcomes among FSWs [29–32].

Elsewhere, we have reported substantial reductions in HIV and STI prevalence among FSWs (2005 to 2011) in the four IBBA districts surveyed [41]. The violence intervention programme described herein began in 2006 and was specifically targeted at violence by the police, strangers and clients, as these were the perpetrators that FSWs first identified as the most problematic. In this study, we observed large secular declines in violence between 2007 and 2011 across 14 of 16 districts where PBS were conducted, but these were not associated with exposure to specific elements of the HIV programme at an individual level (peer education, STI services, use of the drop-in centre or attendance at NGO meetings). There are a number of possible interpretations for this finding. For many of the programme elements, coverage among FSWs by 2008 was extremely high (e.g. ever met a peer educator: 89.1% in 2005, 95.3% in 2008, 97.5% in 2011; ever seen a condom demonstration: 80.6% in 2005, 89.8% in 2008, 93.8% in 2011), making it difficult to interpret findings at the individual level. It may also be that to the extent that the programme reduced risk of violence and arrest, it did so through elements of the programme not monitored through the IBBA and PBS, such as the crisis intervention system, the training of police officers and general advocacy work around sex worker's rights. Alternatively, it may be that women who are more vulnerable to violence or who have experienced police arrest tend to use drop-in centres and other services, which may invert associations. Either way, the reduction in both reported HIV/STI prevalence [41] and in reported violence, arrests and harassment is encouraging and suggests that it is possible to address these structural factors as part of HIV prevention programming.

We also observed significant reductions in reported alcohol use over time. This was surprising, as alcohol reduction was not explicitly included in the prevention programme. Elsewhere, several studies have reported a consistent and positive association between alcohol use and sexual violence experienced by FSWs [9], with alcohol use potentially increasing HIV/STI risk through unprotected sex [45], increased “risky” sex [18] and increased violence risk [45]. As violence, alcohol use and HIV/STI vulnerability are often entwined, it may be that the creation of a more enabling environment and the empowerment of FSWs through community mobilization, support to victims and other structural activities [35] may have led to the reduction in the need of women in these districts to drink alcohol as a (maladaptive) coping mechanism [9].

We observed significant associations between violence by non-partners and HIV prevalence. We also found violence was strongly associated with variables contributing to increased HIV vulnerability, including STI infection, reduced condom use with clients, reduced condom self-efficacy with clients, anal sex with a client and alcohol use in the past week [17, 46]. Although the direct transmission of HIV during rape undoubtedly occurs, multiple, indirect mechanisms are likely important too in increasing vulnerability [3]. Our mediation analyses suggested that alcohol use and STI infection were potentially important in mediating the effect between non-partner violence and HIV infection, but other unmeasured confounders may also be important.

The strong association in this study between being arrested in the past one year and HIV infection was likewise surprising, given that less than 1% of participants reported sexual or physical violence by the police. It is likely therefore that this effect is mediated by indirect factors, some of which were not captured by our quantitative surveys. For example, women who are arrested may be under increased financial pressure to raise bail monies and pay legal and court fees, and this may in turn lead to reduced condom use with clients and/or an increased client load [13, 26]. There are also likely unmeasured confounding variables, as women who are arrested are usually more likely to be working in less safe environments and police harassment might also supress the effectiveness of key programme components such as peer outreach and condom distribution [4, 47]. Mediation analyses again suggested that alcohol use and STI infection may be important on the pathway between arrest and HIV prevalence.

This study had several limitations. Because the analysis examining associations between non-partner violence or arrests and HIV was cross-sectional and used prevalence rather than incidence data, it is not possible to ascertain the causality between violence/arrests and HIV/STI prevalence, especially with a lifelong infection such as HIV [48]. Likewise, it is not possible to determine the directionality of associations between violence/arrests and alcohol use: women who are drunk may be more likely to be arrested or experience violence from clients or others, due to lewd or unruly behaviour [13] and/or women may use alcohol following violence exposure or police arrest, as a coping mechanism for the stress experienced [9]. Further studies are needed to better understand these pathways. The lack of control districts makes it difficult to attribute the reductions in violence, harassment, arrests and alcohol use to programme impact. It is possible that these temporal changes were instead due to chance or reflected naturally occurring trends, although the consistency of findings across multiple districts makes this less likely. The violence questions were relatively simple and reporting bias may have resulted in under-reporting of sensitive topics, such as violence or alcohol use, and over-reporting of others, such as condom use. In addition, the combined violence variable (recent sexual and/or physical violence by a non-partner) used different time references (past 12 months vs. past 6 months, respectively).

Despite these limitations, the findings have several important policy implications. Our findings suggest that it is possible to address violence, police arrest and alcohol use among FSWs through a comprehensive HIV prevention programme. Additional research is needed to better demonstrate, longitudinally, the potential impact of community mobilization interventions in reducing violence from partners of all types and police abuse, as well as to explore the impact of IPV on FSWs’ HIV/STI risk and infection. Ongoing research will assess the cost of the violence component of the Avahan programme, with the aim of informing future budgeting and planning. It will be important for future research to include mental health questions and questions around debt bondage, to try to understand the contribution of depression, anxiety and debt on the pathways between violence/arrests and HIV vulnerability.

## Conclusions

In summary, our findings suggest that, as well as being a violation of women's rights, non-partner violence and police arrest are independently associated with increased HIV and STI prevalence among FSWs. FSW exposures to violence are multiple and pervasive, with important impacts on the physical and emotional health of women who sell sex. The incorporation of FSW empowerment and violence prevention elements into HIV prevention programming is both feasible and effective.

## Supplementary Material

Declines in violence and police arrest among female sex workers in Karnataka state, south India, following a comprehensive HIV prevention programme
