# Supplementary material for: Declines in violence and police arrest among female sex workers in Karnataka state, south India, following a comprehensive HIV prevention programme
Source: J Int AIDS Soc. 2015 Oct 16;18(1):20079. doi: 10.7448/IAS.18.1.20079 (PMC4609649; doi:10.7448/IAS.18.1.20079)
Supplement: Declines in violence and police arrest among female sex workers in Karnataka state, south India, following a comprehensive HIV prevention programme [file JIAS-18-20079-s001.pdf]

Supplementary Table 1. Temporal trends of violence, condom use and condom self-efficacy among Female Sex workers in 4 districts in Karnataka where IBBA surveys were conducted:

	2007 (R1)		2008 (R2)		2009 (R3)		2011 (R4)		Chi2 p value (R1 vs. R4)
	**N**	**%**	**N**	**%**	**N**	**%**	**N**	**%**	
Raped past one year[1]									
**Overall (4 IBBA districts)**	**988**	**31.2**	**1121**	**12.8**	**962**	**15.2**	**1489**	**10.5**	**<0.001**
Bangalore	180	29.4	414	12.1	237	18.1	709	11.3	<0.001
Bellary	307	34.5	275	9.1	258	13.6	303	10.6	<0.001
Belgaum	255	24.7	259	15.8	231	17.3	256	9.4	<0.001
Shimoga	246	35.0	173	16.2	236	11.9	223	9.4	<0.001
Condom use last client[2]									
**Overall (4 IBBA districts)**	**988**	**72.5**	**1121**	**79.8**	**962**	**71.4**	**1489**	**84.2**	**<0.001**
Bangalore	180	73.3	414	79.5	237	84.4	709	83.4	0.002
Bellary	307	66.8	275	80.4	258	69.8	303	80.9	<0.001
Belgaum	255	82.7	259	88.8	231	81.0	256	90.6	0.009
Shimoga	246	68.3	173	66.5	236	50.8	223	83.9	<0.001
Condom self-efficacy[3]									
**Overall (4 IBBA districts)**	**988**	**59.2**	**1121**	**74.8**	**962**	**72.5**	**1489**	**79.3**	**<0.001**
Bangalore	180	52.2	414	67.9	237	66.7	709	76.4	<0.001
Bellary	307	59.0	275	81.5	258	74.8	303	79.2	<0.001
Belgaum	255	66.7	259	81.9	231	70.1	256	83.6	<0.001
Shimoga	246	56.9	173	69.9	236	78.0	223	83.9	<0.001
Did not use condom because she or partner was drunk past month[4]									
**Overall (4 IBBA districts)**	**988**	**30.9**	**1121**	**14.2**	**962**	**20.1**	**1489**	**17.8**	**<0.001**
Bangalore	180	30.0	414	16.2	237	21.1	709	18.9	0.001
Bellary	307	41.4	275	14.5	258	22.1	303	16.2	<0.001
Belgaum	255	18.8	259	9.7	231	20.8	256	15.6	0.29
Shimoga	246	30.5	173	15.6	236	16.1	223	19.3	0.005

Polling Booth Survey data 2007-2011.

[1]In the past one year, were you ever beaten or otherwise physically forced to have sexual intercourse with someone even though you did not want to? [2]Did your last client use a condom with you? [3]Answering 'no' to: During the past one month, was there a time when you wanted to use a condom during sex but did not because your partner did not want to wear a condom? [4]During the past one month, was there a time when you intended to use a condom with a partner but did not use it because either of you had been drinking alcohol? Data from Polling Booth Surveys from four districts where IBBA surveys were also conducted.

Supplementary Table 2. Sociodemographic and sex work characteristics of female sex workers, and violence by non-partners: Pooled data from 4 districts, IBBA R2 and R3

Characteristic		Beaten or otherwise physically forced to have sexual intercourse by non-partner in past one year			Beaten (hurt, hit, slapped, pushed, kicked, punched, choked, burned) in the past 6 months by non-partner		
		No (n=3670)	Yes (n=239)	P value χ^2 test	No (n=3641)	Yes (n=268)	P value χ^2 test
**Current age (years)**	<25	16.0	15.9	0.087	15.4	23.4	0.0010
	25-29	20.6	27.9		20.7	26.3	
	30-39	42.1	40.7		42.6	35.4	
	40+	21.3	15.5		21.4	15.0	
	Mean	32.6	31.5		32.7	30.3	
**Literacy**	Literate	36.0	36.3	0.93	35.9	37.4	0.65
**Additional income to sex work**	Yes	65.0	57.7	0.05	65.4	51.9	0.0003
**Marital status**	Never married/Devadasi	17.9	18.0	0.12	17.8	19.8	0.0024
	Married	36.5	28.6		36.8	25.3	
	Widowed / divorced / deserted (live w/ partner)	10.5	10.9		10.6	9.0	
	Widowed / divorced / deserted (live alone)	35.1	42.5		34.8	45.9	
**Regular Partner**	Yes	63.0	64.0	0.78	63.3	59.5	0.29
**Children**	0	12.4	17.2	0.18	12.0	22.1	<0.0001
	1-2	56.3	55.3		56.2	57.1	
	3+	21.3	27.6		31.8	20.8	
	Mean	1.59	1.53		1.60	1.36	
**Localite**	Living in birth district	79.1	74.9	0.14	79.3	72.4	0.016
**District**	Belgaum	23.8	24.9	0.82	23.1	35.1	0.0006
	Bellary	24.3	24.5		24.4	23.3	
	Shimoga	24.7	26.5		25.5	15.8	
	Bangalore	27.2	24.1		27.0	25.9	
**IBBA round**	2	51.3	40.5	0.007	49.8	61.0	0.004
	3	48.8	59.5		50.2	39.0	
**Age at sexual debut (years)**	<14	22.0	28.5	0.08	22.1	27.4	0.067
	Mean	15.62	15.33		15.62	15.34	
**Age started sex work (years)**	<20	20.4	26.4	0.040	20.1	31.2	<0.001
	20-24	24.5	29.2		24.7	26.9	
	25-29	24.4	22.2		24.5	20.6	
	30+	30.6	22.3		30.7	21.3	
	Mean	25.76	23.84		25.79	23.57	
**Duration sex work (years)**	0-1	17.5	16.9	0.46	17.0	23.6	0.072
	2-4	32.7	28.5		32.7	29.0	
	5-9	24.0	24.0		24.2	20.4	
	10+	25.9	30.6		26.1	27.1	
	Mean	6.82	7.62		6.89	6.68	
**Usual place of solicitation**	Home	28.7	17.5	0.017	28.6	19.5	<0.001
	Brothel/lodge/dabha	7.5	7.7		6.8	18.0	
	Public places	42.5	49.0		42.7	46.6	
	Phone	21.3	25.7		22.0	16.0	
**Usual place of having sex with clients**	Home	63.1	49.4	0.0008	63.6	43.2	<0.001
	Brothel/lodge/dabha	25.6	37.1		25.1	44.1	
	Public places	11.3	13.5		11.3	12.7	
**Clients per typical week**	1-5	38.5	24.2	<0.001	38.8	21.0	<0.001
	6-9	27.5	27.6		27.8	24.0	
	10+	34.0	48.2		33.4	55.0	
	Mean	9.67	12.94		9.35	17.09	
**Charge for sex with last client**	>250 rupees	46.3	46.2	0.99	46.9	37.0	0.010
	Mean (rupees)	331.65	319.41		334.24	285.40	
**Income typical week sex work**	>2000 rupees	38.2	50.1	0.0007	38.0	51.4	<0.001
	Mean (rupees)	2883.22	3581.63		2853.08	3940.54	
**Ever sex work outside district**	Migrant	12.1	29.1	<0.001	12.4	23.5	0.0001
**Sex work in Mumbai ever**	Yes	2.2	5.5	0.0016	2.1	6.7	0.0001

Data from IBBA rounds 2 and 3, four districts (Bangalore, Belgaum, Bellary, Shimoga).

Supplementary Table 3. Violence by non-partners and associations with HIV/STI risk and prevalence: Pooled data from 4 districts, IBBA rounds 2 and 3.

	Beaten or physically forced to have sex past 1 year by non-partner			Beaten (hurt, hit, slapped, pushed, kicked, punched, choked, burned) in the past 6 months by non-partner		
	No (n=3670)	Yes (n=239)	P value Wald test	No (n=3641)	Yes (n=268)	P value Wald test
Programme Exposure						
**Ever seen a peer educator %**	96.41	95.63		96.43	95.39	
Crude OR	0.81 (0.39, 1.70)		0.58	0.77 (0.40, 1.45)		0.41
Adjusted OR	0.57 (0.27, 1.21)		0.14	0.92 (0.47, 1.79)		0.81
**Ever seen a condom demonstration %**	91.16	93.01		91.20	92.46	
Crude OR	1.29 (0.71, 2.33)		0.40	1.18 (0.72, 1.94)		0.50
Adjusted OR	1.16 (0.64, 2.11)		0.63	1.30 (0.79, 2.13)		0.30
**Ever been to an NGO meeting %**	84.57	88.06		85.13	80.32	
Crude OR	1.35 (0.88, 2.04)		0.17	0.71 (0.50, 1.02)		0.062
Adjusted OR	1.25 (0.82, 1.92)		0.31	0.89 (0.62, 1.28)		0.52
**Ever been to a drop-in centre %**	67.27	67.96		67.32	67.26	
Crude OR	1.03 (0.76, 1.41)		0.85	1.00 (0.75, 1.33)		0.98
Adjusted OR	1.10 (0.79, 1.52)		0.57	0.91 (0.67, 1.24)		0.55
**Member of a FSW collective or peer group %**	51.9	48.9		51.77	50.87	
Crude OR	0.89 (0.65, 1.21)		0.45	0.96 (0.72, 1.28)		0.80
Adjusted OR	0.88 (0.63, 1.23)		0.44	1.04 (0.77, 1.39)		0.80
Alcohol use and self-efficacy						
**Alcohol use past one week %**	19.81	36.24		19.69	36.89	
Crude OR	2.30 (1.68, 3.14)		<0.001	2.38 (1.76,3.23)		<0.001
Adjusted OR	2.29 (1.66, 3.17)		<0.001	2.15 (1.57, 2.96)		<0.001
**Answering 'no' to 'In past month had time when wanted to use a condom with a client but did not' (condom *self-efficacy*) %**	87.76	71.33		87.75	72.57	
Crude OR	0.35 (0.25, 0.48)		<0.001	0.37 (0.27, 0.51)		<0.001
Adjusted OR	0.35 (0.25, 0.50)		<0.001	0.31 (0.22, 0.44)		<0.001
Sexual behaviour and HIV/STI service uptake						
**Ever had anal sex with client %**	11.98	34.84		12.61	24.85	
Crude OR	3.93 (2.83, 5.46)		<0.001	2.29 (1.60, 3.28)		<0.001
Adjusted OR	3.83 (2.68, 5.48)		<0.001	2.65 (1.83, 3.86)		<0.001
**Condom use last sex occasional client[1] %**	94.01	90.65		93.69	94.84	
Crude OR	0.62 (0.39, 0.99)		0.046	1.24 (0.70, 2.21)		0.47
Adjusted OR	0.55 (0.34, 0.89)		0.015	1.19 (0.66, 2.14)		0.57
**Condom use last sex repeat client[2] %**	90.2	82.6		89.90	86.00	
Crude OR	0.52 (0.35, 0.77)		0.001	0.69 (0.46, 1.04)		0.076
Adjusted OR	0.51 (0.33,0.77)		0.002	0.64 (0.42, 0.99)		0.042
**Condom use last sex regular partner[3] %**	36.3	40.7		36.68	35.53	
Crude OR	1.20 (0.87, 1.67)		0.27	0.95 (0.65, 1.39)		0.80
Adjusted OR	0.91 (0.63, 1.31)		0.60	0.59 (0.38, 0.92)		0.020
**Condom use last anal sex client %**	77.54	71.63		0.78	0.63	
Crude OR	0.73 (0.40, 1.33)		0.31	0.47 (0.26, 0.86)		0.014
Adjusted OR	0.80 (0.41, 1.59)		0.53	0.57 (0.30, 1.09)		0.087
**STI clinic past 6 months %**	80.5	86.8		80.88	81.24	
Crude OR	1.59 (1.03, 2.46)		0.037	1.02 (0.71, 1.47)		0.90
Adjusted OR	1.39 (0.89, 2.18)		0.15	1.06 (0.72, 1.57)		0.76
**HIV test ever %**	79.0	82.4		79.38	77.13	
Crude OR	1.25 (0.86, 1.81)		0.24	0.88 (0.63, 1.21)		0.43
Adjusted OR	1.01 (0.69, 1.47)		0.95	1.20 (0.83, 1.74)		0.33
HIV and STI infection						
**HIV %**	12.23	19.42		12.10	20.60	
Crude OR	1.73 (1.21, 2.48)		0.003	1.88 (1.32, 2.69)		<0.001
Adjusted OR	1.72 (1.17, 2.54)		0.006	1.49 (1.03, 2.14)		0.032
**Symptomatic STI past 1 year[4] %**	35.66	64.13		36.08	56.72	
Crude OR	3.23 (2.32, 4.49)		<0.001	2.32 (1.79, 3.02)		<0.001
Adjusted OR	3.19 (2.29, 4.44)		<0.001	2.35 (1.78, 3.10)		<0.001
**Chlamydia[5] %**	6.00	4.16		5.76	7.46	
Crude OR	0.68 (0.24, 1.89)		0.46	1.32 (0.64, 2.71)		0.45
Adjusted OR	1.12 (0.39, 3.26)		0.83	1.29 (0.59, 2.82)		0.52
**Gonorrhoea[5] %**	2.24	7.14		2.04	7.52	
Crude OR	3.35 (1.46, 7.69)		0.004	3.90 (2.04, 7.44)		<0.001
Adjusted OR	4.28 (1.69, 10.83)		0.002	3.66 (1.77, 7.55)		<0.001

**Reactive syphilis %**	6.24	6.55		5.90	11.15	
Crude OR	1.05 (0.56, 2.00)		0.87	2.00 (1.25, 3.21)		0.004
Adjusted OR	1.06 (0.55, 2.06)		0.85	1.69 (1.03, 2.76)		0.037
**High-titre syphilis %**	2.55	2.56		2.29	6.09	
Crude OR	1.00 (0.42, 2.40)		0.99	2.77 (1.41, 5.44)		0.003
Adjusted OR	0.99 (0.42, 2.34)		0.98	2.34 (1.17, 4.68)		0.016

Data from IBBA rounds 2 and 3, four districts (Bangalore, Belgaum, Bellary, Shimoga). Models adjusted for district, IBBA round, place where have sex with clients, age, marital status, additional source of income to sex work, and ever sold sex outside the district (migrant sex worker). [1]Occasional client defined as “client who has come to you only once or a few times but you do not remember their face or do not know them.” [2]Repeat client defined as “client you recognize well, who has come to you repeatedly and you know them.” [3]Regular partner defined as “a main (regular) male sexual partner who does not pay to have sex with you?” [4]Sypmtomatic STI past 12 months defined as vaginal discharge, lower abdominal pain (not diarrhoea/menses) and/or genital ulcer/sore. [5]Data available for round 2 only.
